# Screening significance of systemic immune‐inflammation index (SII) and systemic inflammation response index (SIRI) in coronary heart disease of symptomatic youth

**DOI:** 10.1002/iid3.1369

**Published:** 2024-08-07

**Authors:** Chunxiao Wang, Weihong Yan, Mengmeng Ren, Lin Zhong

**Affiliations:** ^1^ Department of Cardiology Yantai Yuhuangding Hospital Yantai Shandong China

**Keywords:** coronary heart disease, systemic inflammation index, systemic inflammation response index, young adults

## Abstract

**Background:**

The incidence of coronary heart disease (CHD) in youth is rapidly increasing but difficultly recognized in the early stage.

**Methods and Results:**

In this retrospective study, 194 CHD patients under the age of 45 who previously experienced chest pain symptoms and 170 non‐CHD patients were included and demographic data were collected. Systemic inflammation index (SII) and systemic inflammation response index (SIRI) were increased in young CHD patients (*p* < 001). Spearman's correlation analysis showed that both SII and SIRI were negatively correlated with HDL and positively correlated with hypertension, Gensini score, and hsTnI. Logistic regression analysis indicated that SII and SIRI were independently associated with the presence of CHD in youth with chest pain symptoms. The area under the ROC curve (AUC) of the SII model for young CHD patients was 0.805 (0.728−0.869), and the sensitivity and specificity were 0.65 and 0.823, respectively. Meanwhile, the AUC for the SIRI model was 0.812 (0.739−0.872), and the sensitivity and specificity were 0.673 and 0.8022. The calibration curves of both SII and SIRI models are in good agreement with the actual curves. And the decision curves of both models indicated their clinical practicality.

**Conclusion:**

SII and SIRI are independent risk factors for CHD in young adults, which can quickly and effectively identify CHD patients among young adults who have previously experienced chest pain symptoms.

## Graphical abstract

(1) Coronary heart disease in youth is difficultly recognized in the early stage. (2) Systemic inflammation index (SII) and systemic inflammation response index (SIRI) were independently associated with the presence of young CHD. (3) SII and SIRI provide a new method for screening patients with CHD in youth.

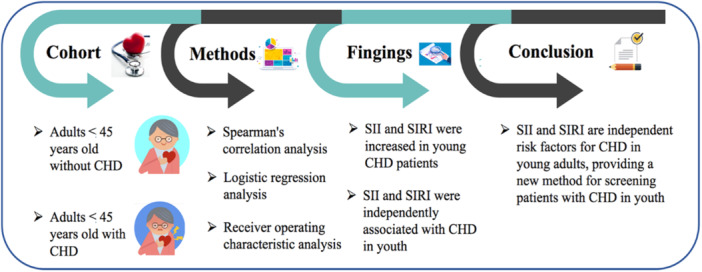

## 1 INTRODUCTION

Coronary heart disease (CHD) is one of the most common causes of sudden cardiac death in young adults today.^1^ However, the incidence of CHD in young adults is rapidly increasing due to obesity, smoking, and unhealthy lifestyles.^2^ Compared with elderly adults with CHD, young patients usually have no obvious symptoms, and the prevalence of myocardial infarction (MI) in young adults is increased due to poor management of risk factors.^3^ Although young patients with MI have a better short‐term prognosis, with in‐hospital mortality and 6‐month mortality rates of only 0.7% and 3.1%, studies have reported a rapid increase in mortality in young patients after 5 years of MI, with mortality rates of more than 15% at 7 years and up to 25%−29% at 15 years.^3^, ^4^ Young patients have a significantly reduced quality of life after MI, which has a much greater impact than older patients.^5^ Therefore, it is important to screen young patients with CHD early for further examination and take effective treatment measures. Although recent studies have shown that a number of indicators, such as lipoprotein (a) and triglyceride‐glucose index, can be used to indicate CHD in young adults, there is no clinical application due to the availability and affordability.^6^, ^7^

Systemic inflammation index (SII) and systemic inflammation response index (SIRI) are novel biomarkers. SII refers to PLT count × neutrophil count/lymphocyte count. Although other indices such as neutrophil‐lymphocyte ratio (NLR) can also reflect the inflammatory state, SII considers the interaction between inflammation and thrombosis and includes the change of platelets, which can better reflect the systemic inflammation.^8^ SIRI, on the other hand, is calculated by absolute numbers of neutrophils, monocytes, and lymphocytes, reflecting the balance between inflammatory responses and immune responses.^9^ Both SII and SIRI are considered to be closely related to the development of tumors and autoimmune diseases.^10^, ^11^, ^12^ Several studies have shown that inflammation plays a key role in the pathogenesis of CHD, which could induce endothelial dysfunction, increase lipoprotein permeability and subendothelial accumulation, exacerbate leukocyte recruitment, and activate platelets.^13^, ^14^ Thus, the severity and prognosis of CHD can be assessed by inflammatory biomarkers. In recent years, SII and SIRI have been shown to be correlated with the occurrence and prognosis of CHD. Increased SII is associated with increased severity of CHD and is positively associated with increased risk of major adverse cardiovascular events.^15^, ^16^ Elevated SIRI is also thought to be associated with an increased risk of overall mortality after MI.^17^ However, the predictive effect of SII and SIRI on CHD in young adults has not been reported. It has been found that young patients with CHD have a stronger inflammatory response compared with elderly CHD patients,^2^ indicating that SII and SIRI may have a better predictive value for CHD of symptomatic youth.

This study aimed to determine the correlation between SII or SIRI and CHD in youth to screen young adults with CHD as early as possible.

## 2 METHODS

### 2.1 Participants

**Figure 1:**
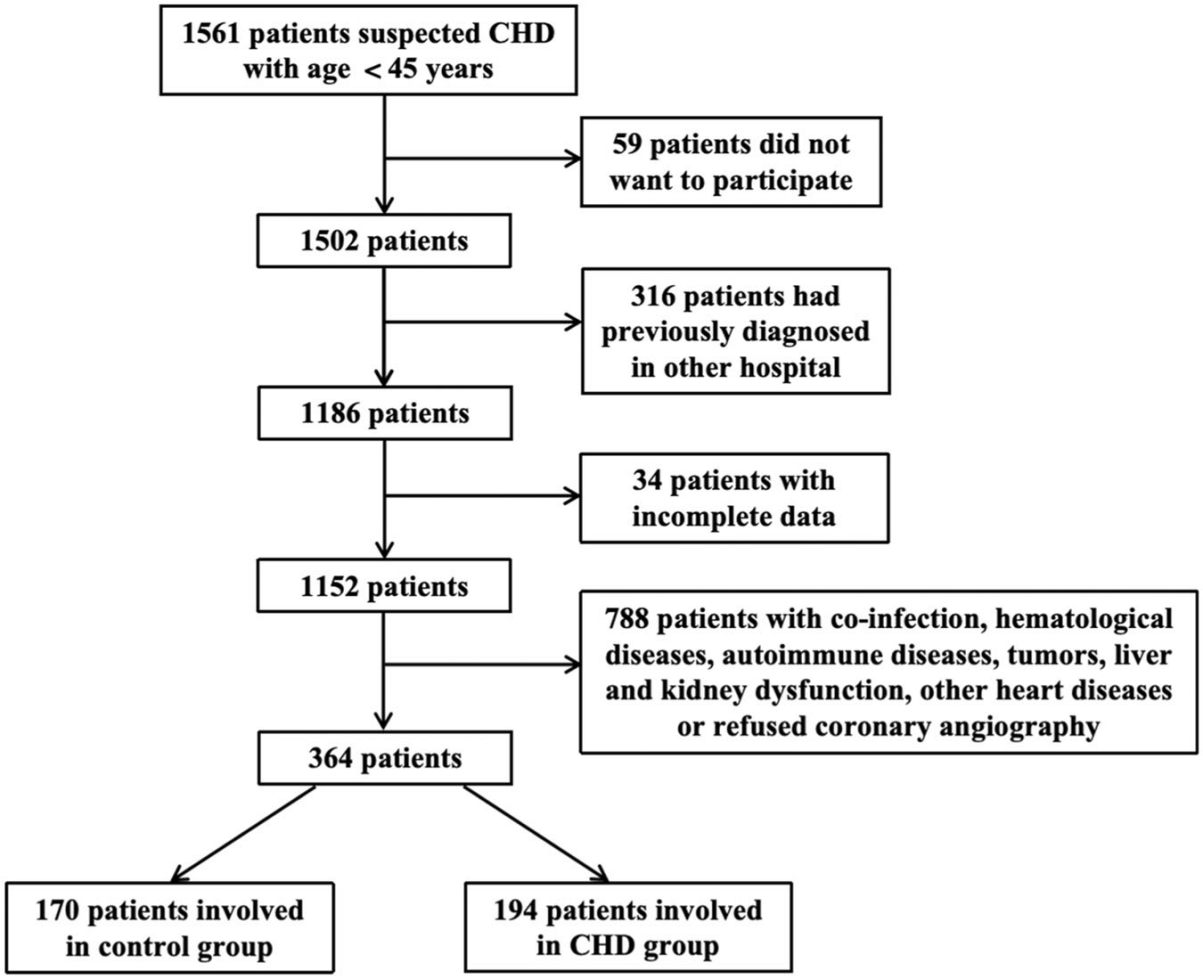
Flowchart shows the study subject screening process. CHD, coronary heart disease.

Three hundred and sixty‐four consecutive young adults under the age of 45 who previously experienced chest pain symptoms admitted to Yantai Yuhuangding Hospital from January 2013 to June 2023 were included to perform the cross‐sectional study. Among them, 194 patients were involved in the CHD patients and 170 in the control group (Figure 1). Patients with the following conditions were included: (1) The patient had at least one previous chest distress or other clinical symptoms related to CHD (Supporting Information S1: Table 1 showed the Chest Pain Questionnaire for details); (2) Have complete admission data and examination results; (3) Coronary angiography was performed and completely recorded. According to the angiographic results, patients with stenosis of more than 50% in at least one major branch of the left main artery, left anterior descending artery, left circumflex artery, or right coronary artery were included in the CHD group. Patients with coronary atherosclerosis, that is, coronary angiography showed less than 50% vascular stenosis, and with normal coronary arteries, that is, coronary angiography showed no abnormalities in the coronary arteries, were included in the control group. Patients with the following conditions were excluded: (1) Infections related diseases, including serious active bacterial or viral infections, chronic persistent infectious diseases, elevated CRP, or serum sedimentation rate; (2) Hematologic disorders；(3) Autoimmune diseases or taking immunosuppressive agents; (4) Active or previous cancer; (5) Severe hepatic and renal dysfunction; (6) Previously diagnosed and treated outside hospital for CHD; (7) Complicated with other heart diseases, including arrhythmia, myocarditis, decompensated heart failure, and so forth; (8) Imperfect clinical data; (9) Refuse to undergo coronary angiography and fail to obtain informed consent. The study protocol conformed to the ethical guidelines of the 1975 Declaration of Helsinki and was approved by the Institutional Clinical Research Ethics Committee (No. 2023‐293). Informed consents were obtained from all patients.

### 2.2 Study variables

A questionnaire was used to collect data on age, sex, medical history, smoking history, and family history. Five milliliters of fasting venous blood was collected from all patients before treatment. Hemoglobin (HGB), white blood cell count (WBC), neutrophil count, lymphocyte count, platelet count, and neutrophil (PLT) were measured using an automatic hematology analyzer (Sysmex). LDH, total cholesterol (TC), triglyceride (TG), high‐density lipoprotein (HDL), low‐density lipoprotein (LDL), lipoprotein a, homocysteine, and troponin I (TnI) were detected by Stream automatic biochemical analyzer (Steam). Color Doppler echocardiography was perfected within 24 h after admission by a cardiac sonographer using a Philips EPIQ 7C color Doppler ultrasound machine (Philips Healthcare Royal Philips Electronics) to collect left ventricular ejection fraction. Coronary angiography was performed in all patients. Six individual positions of the left coronary artery (anteroposterior head position, left head position, spider position, anteroposterior foot position, right foot position, and right head position) and two individual positions of the right coronary artery (left anterior oblique and right anterior oblique) were routinely given to determine the location and degree of coronary artery lesions. Coronary angiography results were analyzed by two interventionalists.

SII and SIRI calculated as previously described^18^: SII = platelet count × neutrophil count/monocyte count; SIRI = neutrophil count × lymphocyte count/monocyte count. The Gensini score was used to quantify the severity of CAD and its specific scoring rules are as follows^19^: the degree of stenosis was based on the most severe site, with a stenosis diameter of <25% for 1 point, 25% ≤ diameter <50% for 2 points, 50% ≤ diameter <75% for 4 points, 75% ≤ diameter <90% for 8 points, 90% ≤ diameter <99% for 16 points, and ≥99% for 32 points. According to different coronary artery branches, the above scores were multiplied by the corresponding coefficients: left main artery disease score multiplied by 5; proximal left anterior descending artery multiplied by 2.5, middle segment score multiplied by 1.5, distal segment score multiplied by 1; first diagonal artery multiplied by 1, second diagonal artery multiplied by 0.5, proximal left circumflex artery multiplied by 2.5, middle, distal, and posterior descending artery multiplied by 1, posterior collateral artery multiplied by 0.5, and proximal, middle, distal, and posterior descending right coronary artery multiplied by 1. The sum of the scores at each lesion site is the total score of the degree of coronary artery stenosis.

### 2.3 Calculation of study sample size

At the beginning of the study, we calculated the sample size by pre‐experiment, that is, 20 patients were respectively collected from the control and CHD group. Pass15 software was applied to calculate the sample size according to SIRI and SII data, respectively. Set the test level *α* = .05, power 1‐β = 0.8. The results showed that the SII index was 527.85 ± 173.82 in the CHD group and 599.04 ± 239.55 in the control, by which calculated the sample size was 137 patients in both groups. The SIRI index was 0.89 ± 0.48 in the CHD group and 1.03 ± 0.31 in the control, and the estimated sample size required was 132 patients in each group. Therefore, 132 samples per group was chosen as the minimum sample size.

### 2.4 Statistical analysis

Baseline characteristics were tested for normality of distribution. Quantitative variables are described as $\bar{x}$ ± s according to the normality test, *t*‐test is used to analyze the difference when the variance is homogeneous, and *t*'‐test is used to analyze the difference when the variance is heterogeneous. Quantitative variables that did not meet normality were described by median and quartiles M (P25−P75), and differences were analyzed by rank sum test. Qualitative variables were described by frequency and constituent ratio using the chi‐squared test when the expected value of the lattice was greater than or equal to 5; corrected chi‐squared when the expected value of the lattice was greater than or equal to 1 and less than 5; and Fisher exact probability method when the expected value of the lattice was less than 1. Spearman's correlation test was used to analyze the correlation between SII, SIRI, and other variables. Confidence intervals (CIs) and standard deviations of correlation coefficients were calculated based on bootstrap (*n* = 1000) resampling. Multivariate logistic regression analyses were performed to examine the association between SII, SIRI, and CHD in young adults, with 95% CIs and odds ratios (ORs). ROC curve (receiver operating characteristic curve) was used to analyze the predictive value of SII, SIRI, and model for CHD in young adults. The calibration curve was obtained by the H‐L (Hosmer–Lemeshow) test to assess the calibration of the prediction model. Decision curve analysis was applied to assess the clinical application value of the prediction model.^20^ All statistical analyses were performed using R software 4.2.3, *p* ≤ .05 (two‐sided) indicated a significant difference.

## 3 RESULTS

### 3.1 Demographic

A total of 364 samples were collected in this study, including 194 young patients in the CHD group and 170 patients in the non‐CHD group. The differences between the two groups were analyzed, as shown in Table 1. The prevalence of hypertension and diabetes was higher in the CHD young patients. In addition, WBC, neutrophil, monocyte, TG, and TnI levels were higher, while HDL levels were decreased in the young CHD group. We also observed that both SII and SIRI were significantly higher in young adults with CHD than in those without CHD (SII: 506.26 [388.04−782.37] vs. 404.46 [306.00−521.46], *p* < .001; SIRI: 1.03 [0.69−1.49] vs. 0.73 [0.54−0.96], *p* < .001).

**Table 1:** Comparison of baseline data between the two groups.

Variables	Non‐CHD group	CHD group	*z*/*χ* ^2^/*t*	*p* Value
Age, years	40.00 (38.00−43.00)	41.50 (38.00−43.00)	0.936	.349
Male, *n* (%)	147 (86.5)	177 (91.2)	2.105	.147
Hypertension, *n* (%)	63 (37.1)	100 (51.5)	7.691	.006*
Diabetes, *n* (%)	8 (4.7)	38 (19.6)	18.175	<.001*
Family history, *n* (%)	29 (17.1)	27 (13.9)	0.687	.407
Smoking, *n* (%)	70 (41.2)	84 (43.3)	0.167	.683
Drug use on admission				
Antihypertension, *n* (%)	35 (20.6)	63 (32.5)	6.506	.011*
Antidiabetes, *n* (%)	4 (2.4)	23 (11.9)	11.914	.001*
Antihyperlipidemia, *n* (%)	25 (14.7)	33 (17.0)	0.359	.549
Gensini score	0.00 (0.00−2.50)	58.50 (40.00−97.00)	10.378	<.001*
HGB, g/L	156.00 (149.00−160.00)	156.5 (147.00−164.00)	0.918	.358
WBC, ×10^9^/L	5.96 (4.95−7.32)	6.89 (5.80−8.24)	2.707	.007*
Neutrophil, ×10^9^/L	3.46 (2.68−4.61)	4.24 (3.45−5.10)	3.233	.001*
Lymphocyte, ×10^9^/L	1.94 (1.74−2.39)	1.96 (1.58−2.39)	0.443	.658
Monocyte, ×10^9^/L	0.42 (0.32−0.51)	0.48 (0.40−0.57)	2.743	.006*
PLT, ×10^9^/L	234.20 ± 45.85	245.00 ± 59.20	1.187	.237
LDH, U/L	175.00 (157.00−206.50)	179.50 (161.00−205.00)	0.557	.577
TC, mmol/L	4.76 (4.30−5.49)	4.76 (4.13−5.43)	0.270	.787
TG, mmol/L	1.28 (1.05−1.79)	1.59 (1.25−2.28)	2.713	.007*
HDL‐C, mmol/L	1.26 (1.05−1.38)	1.10 (0.94−1.25)	2.770	.006*
LDL‐C, mmol/L	3.12 ± 0.90	3.06 ± 1.04	0.334	.739
LP (a), mg/L	91.00 (38.00−237.50)	123.00 (45.00−304.00)	1.093	.275
HCY, μmol/L	12.00 (10.35−13.50)	12.25 (10.40−15.10)	0.757	.449
hsTnI, pg/mL	1.30 (0.20−4.65)	4.45 (1.70−18.90)	4.714	<.001*
EF, %	67.00 (63.00−70.00)	66.00 (63.00−69.00)	0.845	.398
SII	404.46 (306.00−521.46)	506.26 (388.04−782.37)	3.722	<.001*
SIRI	0.73 (0.54−0.96)	1.03 (0.69−1.49)	3.947	<.001*

Abbreviations: EF, ejection fraction; HCY, homocysteine; HDL‐C, high‐density lipoprotein cholesterol; HGB, hemoglobin; hsTnI, high‐sensitivity troponin I; LDH, lactate dehydrogenase; LDL‐C, low‐density lipoprotein cholesterol; LP (a), Lipoprotein a; PLT, platelets; SII, systemic inflammation index; SIRI, systemic inflammation response index; TC, total cholesterol; TG, triglycerides; WBC, white blood cell.

* Statistical significance.

### 3.2 Correlation of SII and SIRI with measured parameters

Spearman's correlation analysis was performed between SII or SIRI and parameters in demographic, respectively. The results showed that both SII and SIRI were negatively correlated with HDL and positively correlated with hypertension, Gensini score, and hsTnI (Table 2).

**Table 2:** Spearman's correlation analysis between SII or SIRI and parameters.

Variable	SII			SIRI		
	*r* (95% CI)	SE	*p*	*r* (95% CI)	SE	*p*
Age	−0.037 (−0.206 to 0.130)	0.001	.654	−0.038 (−0.202 to 0.116)	0.001	.647
Male	−0.061 (−0.188 to 0.093)	0.001	.461	−0.025 (−0.169 to 0.131)	0.001	.763
HBP	0.277 (0.116 to 0.441)	0.001	.001*	0.292 (0.133 to 0.438)	0.003	<.001*
Diabetes	0.039 (−0.111 to 0.194)	0.001	.634	0.074 (−0.103 to 0.235)	0.001	.371
Smoking	0.104 (−0.056 to 0.265)	0.001	.207	0.107 (−0.059 to 0.260)	0.002	.196
GS	0.210 (0.046 to 0.370)	0.002	.010*	0.226 (0.078 to 0.366)	0.001	.005*
HGB	0.090 (−0.077 to 0.243)	0.002	.275	0.136 (−0.021 to 0.295)	0.002	.097
TC	−0.110 (−0.278 to 0.057)	0.002	.180	−0.106 (−0.268 to 0.069)	0.000	.197
TG	0.011 (−0.155 to 0.184)	0.001	.890	0.065 (−0.081 to 0.235)	0.001	.429
HDL‐C	−0.168 (−0.315 to −0.011)	0.001	.040*	−0.246 (−0.380 to −0.084)	0.002	.003*
LDL‐C	−0.004 (−0.173 to 0.165)	0.001	.963	−0.063 (−0.230 to 0.105)	0.000	.447
LP (a)	0.132 (−0.037 to 0.297)	0.001	.108	0.121 (−0.058 to 0.271)	0.000	.140
HCY	0.118 (−0.050 to 0.270)	0.004	.150	0.129 (−0.032 to 0.276)	0.003	.118
hsTnI	0.191 (0.031 to 0.348)	0.001	.020*	0.228 (0.063 to 0.375)	0.003	.005*
EF	0.002 (−0.165 to 0.179)	0.001	.981	−0.056 (−0.224 to 0.113)	0.001	.500

Abbreviations: EF, ejection fraction; GS, Gensini score; HBP, hypertension; HCY, homocysteine; HDL‐C, high‐density lipoprotein cholesterol; HGB, hemoglobin; hsTnI, high‐sensitivity troponin I; LDH, lactate dehydrogenase; LDL‐C, low‐density lipoprotein cholesterol; LP (a), lipoprotein a; PLT, platelets; SII, systemic inflammation index; SIRI, systemic inflammation response index; TC, total cholesterol; TG, triglycerides; WBC, white blood cell.

* Statistical significance.

### 3.3 Logistic regression analysis for SII and SIRI

**Figure 2:**
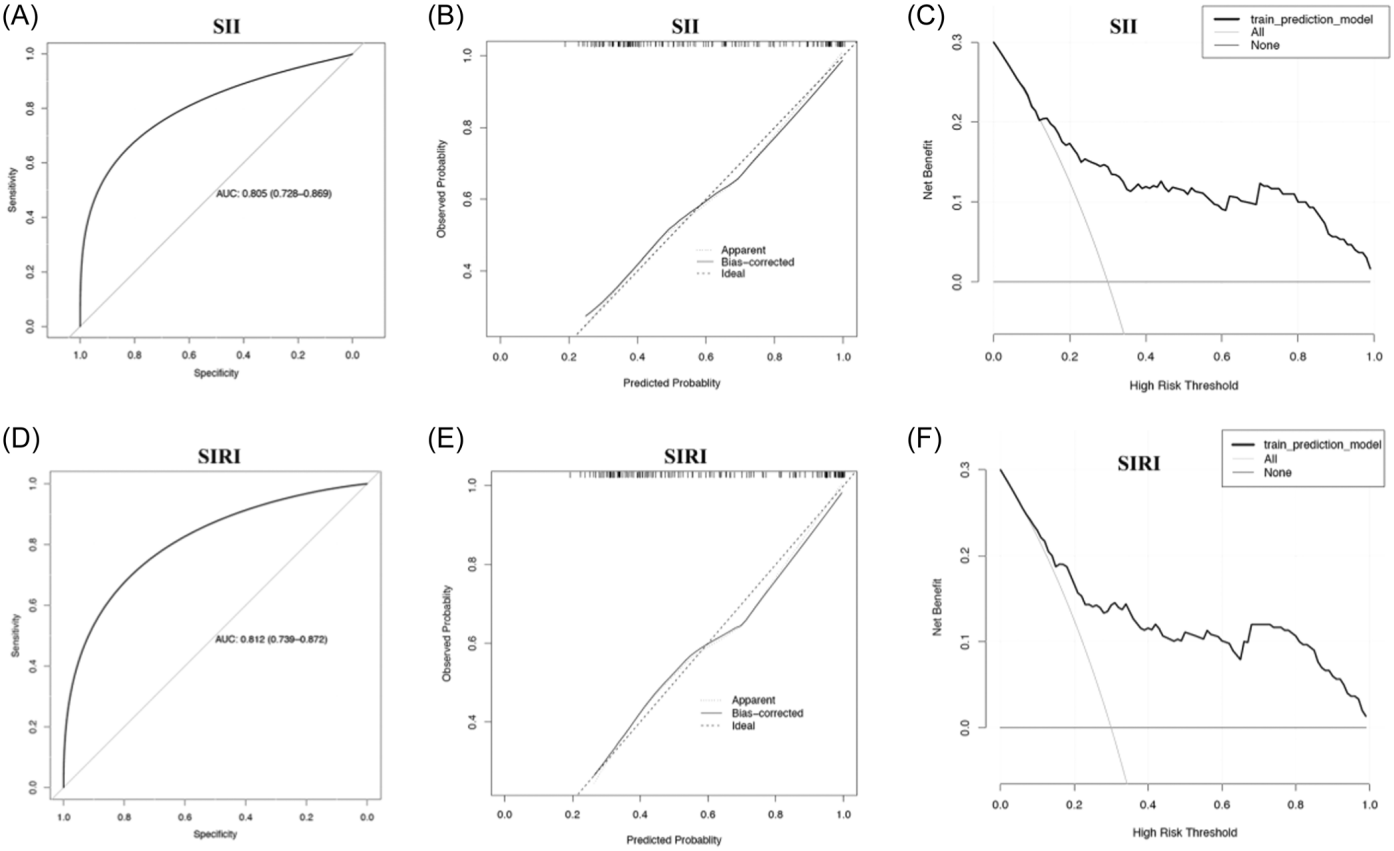
ROC curve, calibration curve, and decision curve of SII and SIRI model. (A and D) Show the ROC curve of SII and SIRI model; (B and E) show the calibration curve of SII and SIRI model; (C and F) show the decision curve of SII and SIRI model. ROC, receiver operating characteristic; SII, systemic inflammation index; SIRI, systemic inflammation response index.

SII and SIRI, as important indicators in this study, have a strong correlation (*r* = .767). When placed in the unified model, the prediction effect will be poor due to multicollinearity. Therefore, multivariate logistic models were constructed for SII and SIRI, respectively to evaluate their prediction effect on CHD. Manually remove variables such as neutrophils, monocytes, and WBCs that are strongly correlated with SII and SIRI. Then, factors (including hypertension, diabetes, Gensini score, TG, HDL‐C, hsTnI, SII, and SIRI) that differed between the control and CHD groups were used to construct the model by stepwise method to select the significant univariate parameters. Finally, diabetes and hsTnI were selected to construct logistic models with SII or SIRI. In the logistic model adjusted for diabetes and hsTnI, SII was an independent associated factor for CHD (Table 3). The area under the ROC curve (AUC) for the SII model was 0.805 (0.728−0.869), and the sensitivity and specificity were 0.656 and 0.823, respectively (Figure 2). Next, the predictive probability threshold in the model was determined by the ROC curve of the multivariate logistic regression model for SII, and univariate logistic regression analysis according to the classification of the threshold showed that when the predictive probability of the SII model was above the threshold, the risk of developing CHD was 8.727 times higher than that below the threshold (OR [95% CI]: 8.727 [3.968−19.196]). At the same time, elevated SIRI is also an associated factor for CHD. For each unit increase in the SIRI index, the risk of CHD changed to 3.753‐fold (OR = 3.753) (Table 4). Meanwhile, the AUC for the SIRI model was 0.812 (0.739−0.872), and the sensitivity and specificity were 0.673 and 0.8022 (Figure 2). When the predicted probability of the SIRI model was above the threshold, the risk of developing CHD was 12.235 times higher than that below the threshold (OR [95% CI]: 12.235 [4.990−29.998]). The calibration curves of both SII and SIRI models are in good agreement with the actual curves. Meanwhile, within the threshold range of 10%−100%, the decision curves of both models were located above the two extreme value lines, indicating their clinical practicality (Figure 2). The above results showed that SII and SIRI models were effective in predicting CHD in the youth.

**Table 3:** Multivariate logistic regression of SII and CHD.

Variables	*β*	SD	*Z*	*p*	OR (95% CI)
Intercept	−1.892	0.536	3.528	< .001*	0.151 (0.050−0.409)
Diabetes	3.188	1.055	3.023	.003*	24.232 (4.628−447.904)
hsTnI	0.066	0.023	2.797	.005*	1.068 (1.027−1.126)
SII	0.003	0.001	3.074	.002*	1.003 (1.001−1.005)

Abbreviations: CHD, coronary heart disease; hsTnI, high‐sensitivity troponin I; SD, standard deviation; SII, systemic inflammation index.

* Statistical significance.

**Table 4:** Multivariate logistic regression of SIRI and CHD.

Variables	*β*	SD	*Z*	*p*	OR (95% CI)
Intercept	−1.615	0.468	3.453	.001*	0.199 (0.075−0.473)
Diabetes	3.105	1.504	2.947	.003*	22.307 (4.271−411.824)
hsTnI	0.060	0.022	2.712	.007*	1.062 (1.024−1.117)
SIRI	1.322	0.451	2.932	.003*	3.753 (1.676−9.780)

Abbreviations: CHD, coronary heart disease; hsTnI, high‐sensitivity troponin I; SD, standard deviation; SII, systemic inflammation index; SIRI, systemic inflammation response index.

* Statistical significance.

## 4 DISCUSSION

Our study found that SII and SIRI were elevated in young adults with CHD. Both SII and SIRI were negatively correlated with HDL and positively correlated with hypertension, Gensini score, and hsTnI. SII and SIRI were independently associated factors for young CHD patients. ROC curve analysis of SII and SIRI showed that SII was more effective in predicting CHD in the youth. The SII and SIRI model (composed of SII or SIRI, diabetes, and hsTnI) have high sensitivity and specificity, which can effectively identify CHD among young adults with previous chest pain symptoms.

Different studies have different age boundaries for CHD in youth. MI has been shown to account for a significant proportion of heart disease in youth under 45 years of age. And significant differences in the incidence of cardiovascular risk factors can be observed at the age cut‐off of 45 years. Multiple cardiovascular risk factors, including blood pressure, dyslipidemia, and diabetes mellitus, were less prevalent in young adults under 45 years old, whereas the incidence of these risk factors increased exponentially beyond 45 years of age. These young patients without risk factors are easy to ignore the occurrence of CHD. At the same time, study has shown that a stronger inflammatory response exist in patients with CHD under 45 years of age, which considered that inflammation‐related SII and SIRI may have a better predictive value in people under 45 years of age.^2^ Therefore, the age cut‐off below 45 years was set in this study.

Young patients with CHD usually also have traditional risk factors. Studies have shown that smoking history, hypertension, diabetes, dyslipidemia, and obesity are closely related to the incidence of CHD.^21^, ^22^ In recent years, the role of new risk factors has also been confirmed in young adults with CHD. It has been shown that 67% of young patients have elevated serum lipoprotein a which is closely related to the occurrence of CHD.^23^ Meanwhile, the content of serum homocysteine was also significantly increased in young CHD patients, and hyperhomocysteinemia is positively associated with CHD in the absence of other risk factors.^24^ In this study, we found that hypertension and diabetes was higher in the CHD young patients than elderly. In addition, WBC, neutrophil, monocyte, TG, and TnI levels were higher, while HDL levels were decreased in the young CHD group.

Among the cases of CHD, the proportion of young patients reaches 3%−4%.^25^, ^26^ And the proportion of sudden death cases in adolescents with cardiac symptoms is not low.^27^ Because asymptomatic young patients rarely go to the hospital for relevant examinations, the proportion of young adults with CHD may be seriously underestimated.^28^ Therefore, it is important to find common and simple biomarkers for early screening of CHD patients in the young population. At present, it has been shown that gene scoring, biomarkers, imaging studies, and multiomics data can be used to identify individuals of high risk.^28^ In this study, we calculated SII and SIRI from common blood routine results and found that both were significantly elevated in young patients with CHD, which suggests that SII and SIRI could applied to screen out patients with possible CHD in symptomatic youth for further examination to confirm the diagnosis. Especially for young patients with transient chest pain symptoms or atypical symptoms, the screening of easy access indicators SII and SIRI is more important, which may effectively improve the early detection rate of CHD in young patients and then improve the prognosis of this CHD population. However, multiple confounding factors may influence the screening effect of SII and SIRI on CHD in youth. Infection, renal insufficiency, autoimmune diseases, and tumors are shown to be associated with SII and SIRI.^11^, ^12^ Therefore, the impact of these diseases on SII and SIRI needs to be excluded during clinical screening.

Recently, SII and SIRI were shown to have a close association with cardiovascular diseases (CVD) which can be predictive markers of CVD and all‐cause mortality in the general population.^29^ SII and SIRI are also associated with the prognosis of a variety of CVD, such as heart failure and cardiac valvular disease^30^, ^31^, ^32^ and their role in CHD has also been concerned. Whether it is stable angina or acute coronary syndrome, SII or SIRI is associated with the severity, and they predict major cardiovascular events better than traditional risk factors after coronary intervention.^33^, ^34^, ^35^, ^36^ The Gensini score is a widely used angiographic scoring system to quantify the severity of CAD which was first described in 1975.^37^ The Gensini score is designed to consideration of the severity of the lesion, the cumulative effect of multiple obstructions, and their location.^38^ Currently, the Gensini score has been considered a widely used angiographic scoring system to quantify the severity of CAD. Studies have shown that with the aggravation of the disease, the Gensini score of CHD patients was significantly higher. The area under the curve (AUC) of Gensini score for adverse CHD outcomes could be as high as 0.9350, suggesting that the Gensini score can effictively reflect the severity of coronary artery disease.^19^ In recent years, the Gensini score has also been widely used to assess the severity of coronary artery disease in young CHD patients.^39^, ^40^ In this study, Genesini score was used to indicate the severity of CHD and assess its correlation with SII and SIRI. The result showed that both SII and SIRI have a positive correlation with the Gensini score, that is, with the severity of CHD. At the same time, a logistic model including diabetes, hsTnI, and SII or SIRI was included. Both diabetes and hsTnI are factors that have been reported to be independently associated with CHD,^41^, ^42^ so including diabetes and hsTnI in the model enabled control of the confounding factors. On this basis, we found that SII and SIRI were independently associated factors for CHD in youth. The AUC for the SII model was 0.805 (0.728−0.869), and the sensitivity and specificity were 0.656 and 0.823, respectively. At the same time, elevated SIRI is also an associated factor for CHD. For each unit increase in SIRI index, the risk of CHD changed to 3.753‐fold (OR = 3.753) (Table 4). Meanwhile, the AUC for the SIRI model was 0.812 (0.739−0.872), and the sensitivity and specificity were 0.673 and 0.8022 (Figure 1). Both models suggests that they are effectively screen the predictability of CHD in young adults with previously chest pain.

### 4.1 Study limitations

This study focuses on young people with CHD. However, due to the low incidence and fewer enrolled people, the cognition of SII and SIRI is limited, which needs further verification in the future. In addition, this study is a single‐center retrospective study which may have admission rate bias. We could do a multicenter prospective study when manpower and material resources are met in the next step.

## 5 CONCLUSION

The SII and SIRI were increased in young CHD patients. Both SII and SIRI have important implications for the young population to suffer from CHD. This study focused on the diagnostic significance of SII and SIRI, providing a convenient, simple, and inexpensive method for screening patients with CHD in youth with previously chest pain symptom.

## AUTHOR CONTRIBUTIONS

All authors contributed to the study's conception and design. Chunxiao Wang was involved in the literature search and manuscript preparation for the entire study. Weihong Yan was responsible for data collection. Mengmeng Ren participated in the data analysis. Lin Zhong was involved in the study design and manuscript review for the entire experiment. All authors have read and approved the final version for publication.

## CONFLICT OF INTEREST STATEMENT

The authors declare no conflict of interest.

## ETHICS STATEMENT

The study protocol conformed to the ethical guidelines of the 1975 Declaration of Helsinki and was approved by the Institutional Clinical Research Ethics Committee (No. 2023‐293). Informed consents were obtained from all patients.

## Supporting information

Supporting information.

Supporting information.

## Data Availability

All the data in the current study could be available from the corresponding author upon reasonable request.
