# Supplementary material for: Screening significance of systemic immune‐inflammation index (SII) and systemic inflammation response index (SIRI) in coronary heart disease of symptomatic youth
Source: Immun Inflamm Dis. 2024 Aug 7;12(8):e1369. doi: 10.1002/iid3.1369 (PMC11304894; doi:10.1002/iid3.1369)
Supplement: Supplementary file 1 — Supporting information. [file IID3-12-e1369-s001.docx]

Name： Age：

| **Chest Pain Questionnaire** |
| --- |
| 1. ***What is your gender？***   □ Male □ Female   1. ***What is your previous medical history?*** |
| □ Hypertension □ Diabetes □ Hyperlipidemia □ Others______ |
| 1. ***What is your smoking history？*** |
| □ Never □<1 year □1-6 years □6-10 years □>10years |
| 1. ***Have you ever had any symptoms of chest pain?*** |
| □ Never □ Occasionally □ Often □ Uncertain |
| 1. ***What is the nature of chest pain？*** |
| □ Suffering pain □ Burning pain □ Colic pain □ Tearing pain □Others______ |
| 1. ***Where is the area of your chest pain?*** |
| □ Precordial □ Middle chest □ Shoulder and back □ Upper abdomen □ Toothache □ Others______ |
| 1. ***Is it associated with radiating pain at other sites*** |
| □ No □ Yes |
| 1. ***What is the degree of your chest pain*** |
| □ Severe □ Heavy □ General □ Light |
| 1. ***Is there a trigger for your chest pain attacks?*** |
| □ No □Exercise or physical work □Emotional excitement □Others______ |
| 1. ***What is the duration of each chest pain？*** |
| □ Seconds □ Minutes □ hours □ days □ Others______ |
| 1. Is there any other symptom accompanying the onset of chest pain? |
| □ Palpitate □ Sweating □ Dyspnea □ Feeling of impending death  □ Others______ |
| 1. ***What measures can be taken to alleviate chest pain?*** |
| □ Waiting for the pain to relieve itself □Oral medication______relieve  □ Accompanied by self or family to hospital □ Call emergency call for assistance |
| 1. ***Have you ever visited a hospital for medical treatment?*** |
| □ No □Yes，once arrived_______ Hospital for treatment |
| 1. Have you ever taken antiplatelet drugs? |
| □ No □Yes，please indicate the type and duration of medication used___________ |
| 1. Are there any patients with coronary heart disease among their immediate family members? |
| □ No □ Yes |
